# Measurement of autophagy via LC3 western blotting following DNA-damage-induced senescence

**DOI:** 10.1016/j.xpro.2022.101539

**Published:** 2022-07-11

**Authors:** Hitomi Yamamoto-Imoto, Eiji Hara, Shuhei Nakamura, Tamotsu Yoshimori

**Affiliations:** 1 Department of Genetics, Graduate School of Medicine, Osaka University, Suita, Osaka 565-0871, Japan; 2 Research Institute for Microbial Diseases (RIMD), Osaka University, Suita, Osaka 565-0871, Japan; 3 Immunology Frontier Research Center (IFReC), Osaka University, Suita, Osaka 565-0871, Japan; 4 Laboratory of Intracellular Membrane Dynamics, Graduate School of Frontier Biosciences, Osaka University, Suita, Osaka 565-0871, Japan; 5 Institute for Advanced Co-Creation Studies, Osaka University, Suita, Osaka 565-0871, Japan; 6 Integrated Frontier Research for Medical Science Division, Institute for Open and Transdisciplinary Research Initiatives (OTRI), Osaka University, Suita, Osaka 565-0871, Japan

**Keywords:** Cell Biology, Cell culture, Cell-based Assays

## Abstract

Senescent cells accumulation is associated with aging and age-related diseases, and recent findings suggest that autophagy, the activity of the intracellular degradation system, decreases during senescence. In this protocol, we detail steps to induce cellular senescence in response to DNA damage, evaluate the senescent state using SA-β-gal staining and western blot for p21, LAMP1, and Lamin B1, and detect autophagy via LC3 western blotting. This protocol can be used in most cell lines and for various types of senescent cells.

For complete details on the use and execution of this protocol, please refer to Yamamoto-Imoto et al. (2022).

## Highlights

• Steps to induce cellular senescence in response to DNA damage
• SA-β-gal staining and western blot for p21, LAMP1, and Lamin B1 to evaluate senescence
• SDS-PAGE and western blot for LC3 to assess autophagic activity
• This protocol can be used in various cell lines to investigate autophagy

## Abstract (editor highlights)

Publisher’s note: Undertaking any experimental protocol requires adherence to local institutional guidelines for laboratory safety and ethics.

## Graphical abstract

**Figure:**
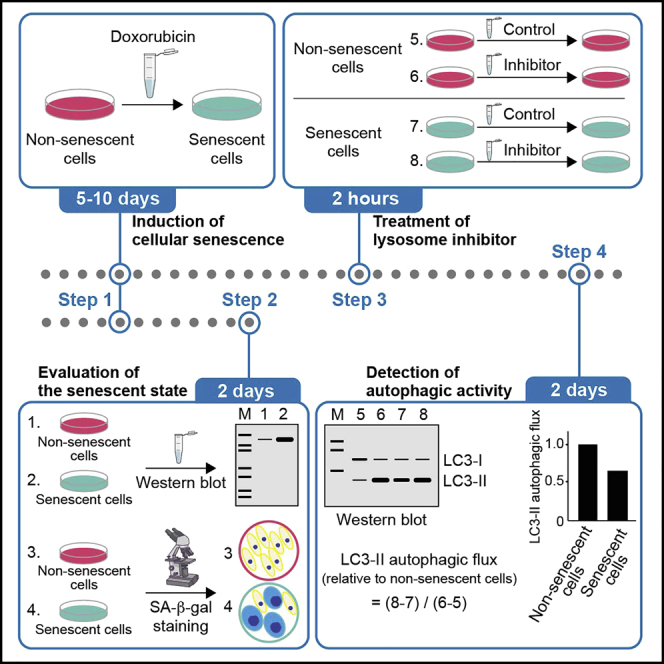

## Before you begin

**CRITICAL:** All materials for cell culture need to be sterile, and all sterile procedures handling cells need to be performed in a Class II biological safety cabinet under standard aseptic techniques.

### Cell preparation

**Timing: 1 day**

1. Passage the proliferative human retinal pigment epithelial cell line (hRPE1 cells) using Trypsin-EDTA solution.
2. Plate the cells to evaluate senescent state.
  a. For senescence-associated β-galactosidase (SA-β-gal) staining.
    i. Dilute the Cellmatrix Type I-C (Fujifilm Wako, 631-00771) 1:10 in ddH2O.
    ii. Put micro cover glass (0.16–0.19 mm thickness) in 35 mm culture dish (Thermo Fisher Scientific, 150460).
    iii. Coat the dish with 2 mL of Cellmatrix Type I-C solution.
    iv. Incubate the dish for 10 min at room temperature.
    v. Wash the dish twice with 2 mL of PBS, and remove the wash solution.
    vi. Plate the cells on the dish at the density of 0.5 × 10^5^ cells per dish.
  b. For SDS-PAGE and Western blot.
    i. Plate the cells on 6-well dish (Nunc™ Cell-Culture Treated Multidishes, Thermo Fisher Scientific, 140675) at the density of 0.5–1.0 × 10^5^ cells per well.
3. Incubate the cells for 24 h at 37°C with 5% CO_2_ in a humidified chamber.

***Note:*** Nomal cells need to be seeded at the density of 0.5 × 10^5^ cells per dish or well 3 days before sample preparation to evaluate the senescent state (step 4) or detection of autophagic activity (step 19) in step-by-step method details.

***Note:*** Bring PBS and culture medium to room temperature (20°C–22°C) before starting cell culture.

### Reagent preparation

#### Doxorubicin stock solution

**Timing: 10 min**

4. Dissolve doxorubicin reagent in ddH_2_O to obtain the stock solution (the concentration is 150 ng/μL). Store the stock solution at ≤−20°C, protected from light.

**CRITICAL:** Doxorubicin reagent causes harmful if swallowed. Also, this may cause asthmatic symptoms if inhaled. Please see the safety data sheet to prevent above (https://labchem-wako.fujifilm.com/sds/W01W0104-2152JGHEEN.pdf). This should be prepared in a Class II biological safety cabinet.

#### Bafilomycin A1 stock solution

**Timing: 10 min**

5. Dissolve bafilomycin in dimethyl sulfoxide (DMSO) to obtain the stock solution (the concentration is 250 μM). Store the stock solution at ≤−20°C, protected from light.

## Key resources table

**Table:** 

REAGENT or RESOURCE	SOURCE	IDENTIFIER
**Antibodies**		

Rabbit polyclonal anti-LC3 (1:1000 dilution)	MBL	Cat#PM036; RRID: AB_2274121
Rabbit monoclonal anti-p21 [EPR3993] (1:1000 dilution)	Abcam	Cat#ab109199; RRID: AB_10861551
Rabbit polyclonal anti-Lamin B1 – Nuclear Envelope Marker (1:1000 dilution)	Abcam	Cat#ab16048; RRID: AB_10107828
Mouse monoclonal anti-LAMP1 (H4A3) (1:1000 dilution)	Santa Cruz Biotechnology	Cat#sc-20011; RRID: AB_626853
Rabbit polyclonal anti-α-Tubulin (1:64000 dilution)	MBL	Cat#PM054; AB_10598496
Peroxidase-AffiniPure Goat Anti-Rabbit IgG (H+L) (1:10000 dilution)	Jackson ImmunoResearch	Cat#111-035-003; RRID: AB_2313567
Peroxidase-AffiniPure Goat Anti-Mouse IgG (H+L) (1:10000 dilution)	Jackson ImmunoResearch	Cat#115-035-003; RRID: AB_10015289

**Chemicals, peptides, and recombinant proteins**		

Dulbecco’s Modified Eagle’s Medium -low glucose-	Sigma-Aldrich	Cat#D6046
Fetal Bovine Serum	Sigma-Aldrich	Cat#F7524
Penicillin-Streptomycin	Sigma-Aldrich	Cat#P4333
Cellmatrix Type I-C	Fujifilm Wako	Cat#631-00771
Trypsin-EDTA solution	Sigma-Aldrich	Cat#T4174
Doxorubicin	FUJIFILM Wako Chemicals	040-21521; CAS RN® : 25316-40-9
Bafilomycin A1	Cayman Chemical	11038; CAS RN® : 88899-55-2
Dimethyl Sulfoxide	Nacalai tesque	09659-14; CAS RN® : 67-68-5
cOmplete, EDTA-free	Roche	11873580001
Ponceau S	Sigma-Aldrich	Cat#24-3860; CAS No. : 6226-79-5

**Critical commercial assays**		

Senescence Cells Histochemical Staining Kit	Sigma	CS0030
Pierce^TM^ bicinchoninic acid Protein Assay Kit	Thermo Fisher Scientific	23225

**Experimental models: Cell lines**		

hRPE cells	Lonza Inc.	00194987

**Software and algorithms**		

Prism v8.4.3	GraphPad software	https://www.graphpad.com/scientific-software/prism/; RRID: SCR_002798
cellSens Standard	Olympus	https://www.olympus-lifescience.com/en/software/cellsens/; RRID: SCR_014551
ImageJ	(Schneider et al., 2012)	https://fiji.sc; RRID: SCR_002285

## Materials and equipment

**Timing: 5 min for culture medium**

**Timing: 15 min for lysis buffer**

**Timing: 30 min for 5× SDS-sample buffer**

**Timing: 10 min for Ponceau S**

**Table:** Culture medium

Reagent	Final concentration	Amount
Dulbecco’s Modified Eagle’s Medium -low glucose-	n/a	500 mL
Fetal Bovine Serum	10%	50 mL
Penicillin-Streptomycin	1%	5 mL
**Total**	**n/a**	**555 mL**

Store at 4°C. The solution is stable for up to 1 month.

***Alternatives:*** Culture medium depends on the type of cells, and purpose of experiment.

**Table:** Lysis buffer

Reagent	Final concentration	Amount
1 M Tris-HCl, pH 7.5	50 mM	2.5 mL
5 M NaCl	150 mM	1.5 mL
Deoxycholic acid	0.25%	0.125 g
Nonidet^TM^ P-40	1%	0.5 mL
500 mM EDTA	1 mM	100 μL
100× cOmplete, EDTA-free	1×	500 μL
100 mM Phenylmethylsulfonyl fluoride (PMSF)	1 mM	500 μL
ddH2O	n/a	to 50 mL
**Total**	**n/a**	**50 mL**

Store at ≤−20°C. The solution is stable for up to 1 year.

***Note:*** Protease inhibitors including cOmplete and PMSF should be added just before use.

***Alternatives:*** Other protease inhibitors and detergents can be used. PhosSTOP^TM^ (04906845001; Roche) can be added to the lysis buffer to assess the expression levels of phosphorylated protein such as Rb, NF-κB, and AMP-activated protein kinase as senescence markers.

**Table:** 5× SDS-sample buffer (pH 6.8)

Reagent	Final concentration	Amount
SDS	10%	10 g
Tris-base	50 mM	0.606 g
Dithiothreitol	250 mM	3.856 g
EDTA	10 mM	0.372 g
Glycerol	30% (v/v)	30 mL
HCl	n/a	Adjust pH to 6.8
ddH20	n/a	to 100 mL
Bromophenol blue	0.1% (w/v)	0.1 g
**Total**	**n/a**	**100 mL**

Store at ≤−80°C. The solution is stable for up to 1 year.

***Note:*** Bring this buffer to room temperature (20°C–22°C) before mixing with samples.

**Table:** Ponceau S

Reagent	Final concentration	Amount
Ponceau S	0.2%	0.4 g
Acetic acid	1%	2 mL
ddH2O	n/a	to 200 mL
**Total**	**n/a**	**200 mL**

Store at room temperature. The solution is stable for up to 1 year.

## Step-by-step method details

### Induction of cellular senescence as response to DNA damage

**Timing: 5 or 10 days**

This step should be carried out at 40%–50% confluency.

1. Dilute the doxorubicin stock solution 1:1000 in culture medium to obtain the doxorubicin solution (the final concentration is 150 ng/mL).
2. Wash each well twice with 2 mL of PBS, and remove the wash solution.
3. Add 2 mL of the doxorubicin solution per well, and maintained at 37°C with 5% CO_2_ in a humidified chamber for 5 or 10 days. No need to change the culture medium during the treatment. Troubleshooting 1.

**Figure 1:**
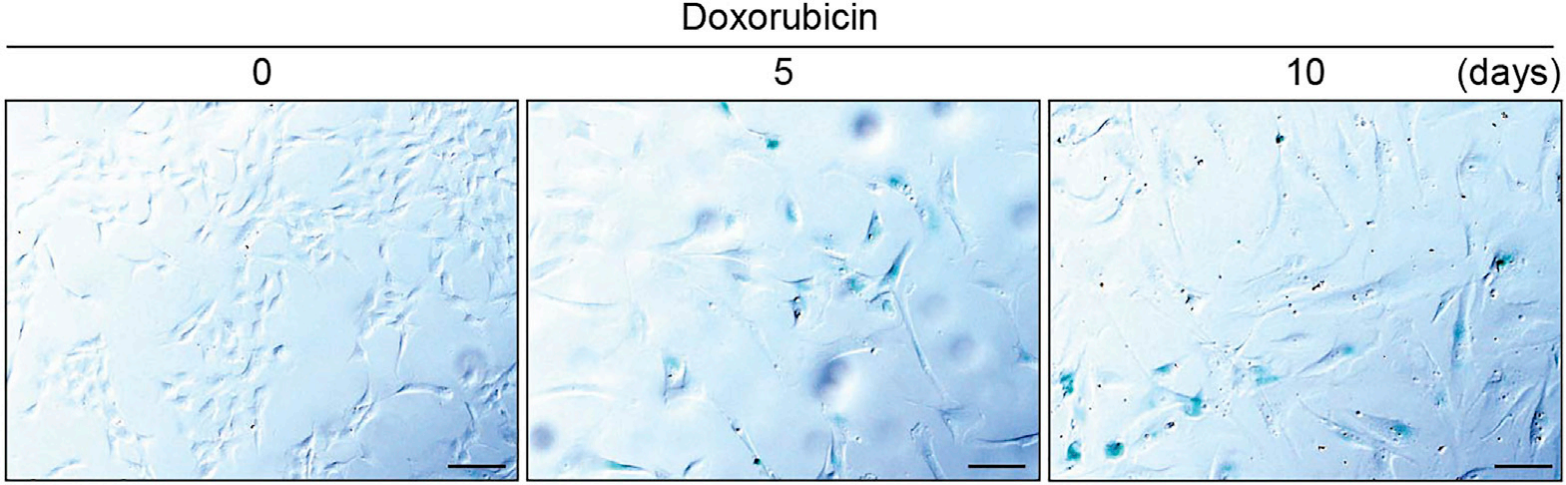
Increased SA-β-gal-positivity in senescent cells Representative staining images of SA-β-gal (colored blue) in non-induced and senescent cells treated with doxorubicin (150 ng/mL) for 5 or 10 days. Scale bar, 100 μM.

**Figure 2:**
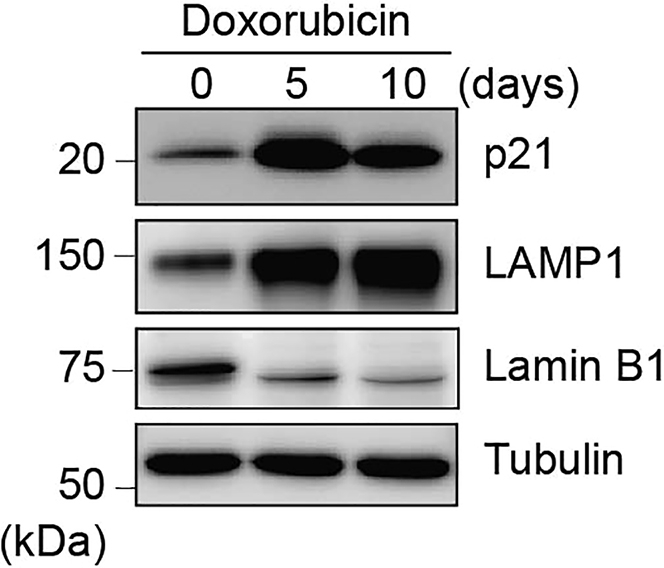
Increased levels of p21 and LAMP1, and decreased expression of Lamin B1 in senescent cells Expression of senescence markers including p21, LAMP1, and Lamin B1 in hRPE1 cells treated with doxorubicin (150 ng/mL) for 5 or 10 days. Representative Western blots from three independent experiments.

**CRITICAL:** Although cellular senescence is induced in cells treated with doxorubicin for 5 days as seen in the senescence markers including cyclin-dependent kinase inhibitor p21, lysosome-associated membrane protein 1 (LAMP1), and Lamin B1 in Figure 2, SA-β-gal positivity is still not fully increased compared with cells treated for 10 days (Figure 1) (Yamamoto-Imoto et al., 2022). Therefore, we recommend to investigate in both situations to decide which is suitable state for your experiments. Duration of doxorubicin treatment can be changed if the possibility remains that alternative time points could lead to differences in your data.

### Sample preparation to evaluate the senescent state

**Timing: 1 h**

Before exploring autophagic activity, senescent state should be determined by SA-β-gal staining and Western blot for p21, LAMP1, and Lamin B1.

***Note:*** Since senescent cells show enlarged cell size (Bent et al., 2016; Druelle et al., 2016), observing using normal microscopy can easily help to determine when to evaluate senescent state. These morphological changes are seen in Figure 1.

4. At 10 days after the treatment of doxorubicin, prepare samples for SA-β-gal staining and Western blot.
  a. Prepare the samples for SA-β-gal staining using Senescence Cells Histochemical Staining Kit (Sigma, CS0030).
    i. Wash each dish with 2 mL of PBS, then remove the wash solution.
    ii. Add 2 mL of 1× Fixation Buffer and incubate for 7 min at room temperature.
    iii. Wash each dish 3 times with 2 mL of PBS.
      **Pause point:** After step 4.a.iii. samples can be stored in PBS at 4°C for a week.
  b. Prepare the samples for Western blot.
    i. Wash each well twice with 2 mL of cold-PBS, then remove the wash solution.
    ii. Add 25 μL of lysis buffer and scrape each well on ice.
      ***Note:*** The amount of lysis buffer depends on the cell number. A suggested amount of lysis buffer is 25–100 μL.
    iii. Pipette 7 times to lyse well, and then transfer to a microcentrifuge tube.
    iv. Centrifugation at 20,000 × *g* for 15 min at 4°C.
    v. Transfer the supernatant to a new microcentrifuge tube.
    vi. Measure the protein concentration using Pierce BCA Protein Assay Kit as described in https://www.thermofisher.com/order/catalog/product/23225.
    vii. Adjust the protein concentration among all samples using lysis buffer and mix well with 5× SDS-sample buffer.
    viii. Heat them at 95°C for 7 min, and then place the tube at room temperature for 5 min.
      **Pause point:** Samples at steps 4.b.v. and 4.b.viii. can be stored at ≤−80°C and ≤−20°C until use, respectively.

### Evaluation of the senescent state

#### SA-β-gal staining

**Timing: 2 days**

5. Transfer the micro cover glass with the fixed cells from step 4.a.iii. into 24-well plate filled with PBS.
6. Remove the PBS.
7. Add 1 mL of Staining mixture including X-gal Solution (Sigma, CS00030).
8. Seal the plate with Parafilm.
9. Incubate the cells at 37°C for 16–20 h without CO_2_.

***Note:*** Appropriate staining time should to be optimized in each types of cell using microscope.

10. Wash the cells three times with 1 mL of PBS.
11. Cover with Mounting Medium with DAPI (VECTASHIELD, H-1200) and observe the cells on DIC and DAPI images with Olympus cellSens Standard Imaging Software using microscope (100× magnification, BX53, Olympus). Count more than 200 DAPI-positive cells manually, and calculate the percentage of SA-β-gal-positive cells per condition in each experiment. Please refer to (Yamamoto-Imoto et al., 2022). Troubleshooting 2.

***Optional:*** Cellular Senescence Plate Assay Kit -SPiDER-βGal (SG05, Dojindo) and Cell Count Normalization Kit (C544, Dojindo) can be useful alternatives to assess SA-β-gal positivity (https://dojindo.com/product/cellular-senescence-plate-assay-kit-spider-aygal-sg05/).

#### SDS-PAGE and western blot

**Timing: 2 days**

For more details, please see Yamamoto-Imoto et al. (2022).

12. Lysates are run on reducing SDS-PAGE gels.

***Note:*** Load 10–20 μg of total protein, but less amount of total protein can be used when senescence is markedly induced.

13. Blot the protein to PVDF membrane and stained with Ponceau S to confirm the proteins are equally blotted among samples.
14. Blocking with 1% skim milk for 30 min at room temperature.
15. Incubated overnight at 4°C in primary antibody.
16. Incubated with appropriate HRP-conjugated secondary antibody at room temperature for an hour.
17. Blots are developed with Immobilon Forte Western HRP Substrate or ImmunoStar LD.
18. Signals are detected in ChemiDocTM Touch Imaging System (Bio-Rad). Troubleshooting 3.

***Note:*** After washing the membrane with TBST and ddH_2_O, dried membrane can be stored at room temperature to reuse for detecting other senescence markers.

**CRITICAL:** Because senescence is highly complex and heterogenous (Gorgoulis et al., 2019), various senescent phenotypes should be confirmed as described in (Kohli et al., 2021). Especially, Cell cycle arrest and mitochondrial dysfunction should be assessed, for example by using Click-iT® EdU Imaging Kit (Invitrogen, C10337) and XF Cell Mito Stress Test Kit that includes Oligomycin, FCCP, and Rotenone/Antimycin A (Agilent Technologies, 103015-100), respectively (Yamamoto-Imoto et al., 2022).

### Detection of autophagic activity

**Timing: 3 h**

Although this protocol describes the specific steps to assess autophagy in DNA damage-induced senescent cells, we have also used this protocol in the replicative senescence model. It is definitely important to compare the expression of LC3-II between the cells treated with or without bafilomycin A1, which is the inhibitor of the fusion of autophagosome and lysosome as described in (Mizushima et al., 2010). Although confirming the LC3 expression level using microscope might be one of the indicators related to the autophagic activity, it still remain unclear that increased levels of LC3-II is due to the upregulation of autophagosome formation or inhibition of autophagic degradation of LC3-II.

19. Dilute the bafilomycin A1 stock solution or DMSO 1:1250 in culture medium to obtain the bafilomycin A1 (the final concentration is 200 nM) or DMSO solution.
20. Wash each well twice with 2 mL of PBS, then remove the wash solution.
21. Add 2 mL of culture medium with bafilomycin A1 or DMSO solution.
22. Incubate the cells for 2 h at 37°C with 5% CO_2_ in a humidified chamber.
23. Prepare the samples by the same method as step. 4.b.
24. Perform SDS-PAGE and Western blot for LC3 as described above. Troubleshooting 4.

***Note:*** Performing SDS-PAGE in 15% gel is better to detect LC3-II.

***Optional:*** Increased levels of Rubicon can be the useful indicator of decreased autophagic activity (Yamamoto-Imoto et al., 2022; Matsunaga et al., 2009).

***Optional:*** Measuring the expression levels of p62, which interact with autophagic substrates, with or without bafilomycin A1 is also used to explore autophagic activity.

## Expected outcomes

**Figure 3:**
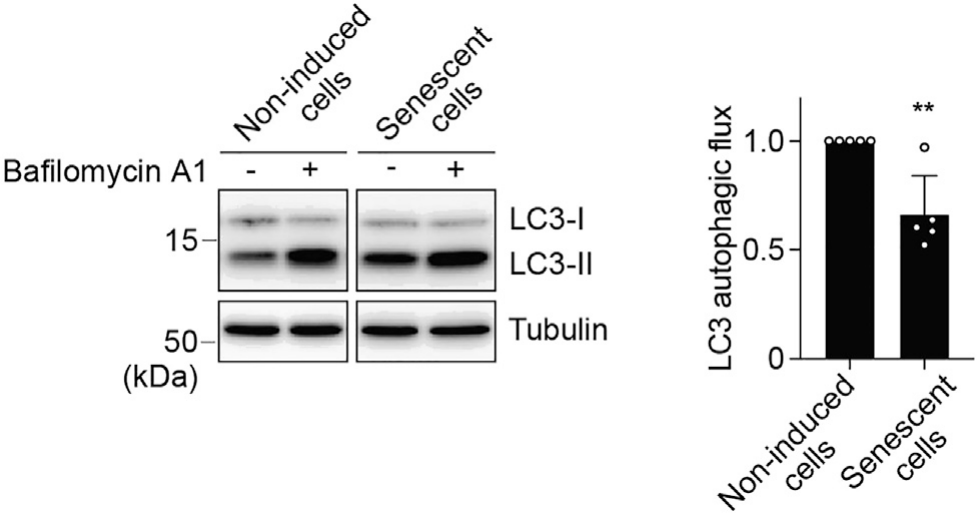
LC3 autophagic flux declines during senescence Autophagic flux, determined by subtracting LC3-II expression in the samples treated with the lysosome inhibitor, bafilomycin A1 (200 nM, 2 h), from the ones without the inhibitor, is decreased in senescent cells treated with doxorubicin for 10 days. Representative Western blots (left) and quantification (right) from five independent experiments. Relative intensity to non-induced cells are presented as the mean ± SD. Statistical analyses were performed using an unpaired t-test.

SA-β-gal positivity is increased in DNA damage-induced senescent cells induced by doxorubicin treatment for 5 or 10 days (Figure 1). In addition, increased levels of p21 and LAMP1, and decreased expression of Lamin B1 can be detected in senescent cells (Figure 2). LC3 autophagic flux, determined by subtracting LC3-II expression in the samples treated with bafilomycin A1 from the ones without the inhibitor, can be decreased in senescent cells compared with non-induced cells (Figure 3).

## Quantification and statistical analysis

Data were quantified using densitometry with ImageJ (https://fiji.sc) (Schneider et al., 2012). Statistical analyses were performed using Prism software (https://www.graphpad.com/scientific-software/prism/). Data are denoted as means ± standard deviation (SD). p values ≤ 0.05 were considered significant (∗), with values ≤ 0.01 designated ∗∗.

## Limitations

Since the long-term treatment of bafilomycin A1 triggers premature senescence as seen in the upregulated levels of p21, p53, and SA-β-gal positivity, and mitochondrial dysfunction determined by increased mitochondrial superoxide (Takenaka et al., 2022), treatment duration of bafilomycin A1 needs to be shorter as possible.

## Troubleshooting

### Problem 1

Poor cells after the doxorubicin treatment for 10 days (step 3).

### Potential solution

Spread cells to be able to maintain a confluence of 80% after the senescence induction. Please note that since DNA-damage drives drastic changes in cell size and morphology, it is not recommended to spread too many cells before the induction of senescence.

### Problem 2

SA-β-gal positivity is not increased after the doxorubicin treatment (steps 4a, 5–11).

### Potential solution

• Optimize the concentration and duration of the doxorubicin treatment in each types of cell.
• Use the appropriate Fixation Buffer, do not use 4% paraformaldehyde to fix the cell. If cells were fixed with 4% paraformaldehyde, it may be hard to see the cells coloring blue even after incubation for 20 h.
• Incubate the cells longer with Staining mixture at 37°C without CO_2_ and observe well using microscope.

### Problem 3

Expression levels of senescent markers determined by Western blot are not altered (steps 4b, 12–18).

### Potential solution

• Confirm the concentration of loading protein and primary antibody in SDS-PAGE and Western blot.
• Other hallmarks of senescent state, such as p16 and senescence-associated secretory phenotype (SASP) (Gorgoulis et al., 2019), need to be addressed.
• Strongly recommend to optimize the concentration and duration of the doxorubicin treatment if both Problem 2 and 3 occurred.

### Problem 4

LC3-II is not accumulated even in the bafilomycin A1-treated cells (steps 19–24).

### Potential solution

• Concentration and appropriate duration of the bafilomycin A1 treatment need to be optimized. Please note that the treatment duration of bafilomycin A1 should be shorter as described in Limitation above.
• Other lysosomal inhibitors such as chloroquine can be used.
• Performing SDS-PAGE in 15% gel is better to detect LC3-II.
• Tandem fluorescent-tagged LC3 is the useful alternative to determine the autophagic activity. Please refer to (Kimura et al., 2007) for complete details.

## Resource availability

### Lead contact

Further information and requests for resources and reagents should be directed to and will be fulfilled by the lead contact, Shuhei Nakamura (shuhei.nakamura@fbs.osaka-u.ac.jp).

### Materials availability

This study did not generate new unique reagents.

## Data Availability

This study did not generate and analyze any original datasets/codes.
